# The HNF1α-regulated lncRNA HNF1A-AS1 reverses the malignancy of hepatocellular carcinoma by enhancing the phosphatase activity of SHP-1

**DOI:** 10.1186/s12943-018-0813-1

**Published:** 2018-02-21

**Authors:** Chen-Hong Ding, Chuan Yin, Shi-Jie Chen, Liang-Zhi Wen, Kai Ding, Shu-Juan Lei, Jin-Pei Liu, Jian Wang, Kai-xian Chen, Hua-liang Jiang, Xin Zhang, Cheng Luo, Wei-Fen Xie

**Affiliations:** 1 Department of Gastroenterology, Changzheng Hospital, Second Military Medical University, 415 Fengyang Road, Shanghai, 200003 China; 2 Drug Discovery and Design Center, CAS Key Laboratory of Receptor Research, State Key Laboratory of Drug Research, Shanghai Institute of Materia Medica, Chinese Academy of Sciences (CAS), Shanghai, 201203 China; 3 Present address: Department of Gastroenterology, Institute of Surgery Research, Daping Hospital, Third Military Medical University, Chongqing, 400042 China

**Keywords:** HNF1α, HNF1A-AS1, Hepatocellular carcinoma, SHP-1, phosphatase activity

## Abstract

**Background:**

Our previous study has demonstrated that hepatocyte nuclear factor 1α (HNF1α) exerts potent therapeutic effects on hepatocellular carcinoma (HCC). However, the molecular mechanisms by which HNF1α reverses HCC malignancy need to be further elucidated.

**Methods:**

lncRNA microarray was performed to identify the long noncoding RNAs (lncRNAs) regulated by HNF1α. Chromatin immunoprecipitation and luciferase reporter assays were applied to clarify the mechanism of the transcriptional regulation of HNF1α to HNF1A antisense RNA 1 (HNF1A-AS1). The effect of HNF1A-AS1 on HCC malignancy was evaluated in vitro and in vivo. RNA pulldown, RNA-binding protein immunoprecipitation and the Bio-Layer Interferometry assay were used to validate the interaction of HNF1A-AS1 and Src homology region 2 domain-containing phosphatase 1 (SHP-1).

**Results:**

HNF1α regulated the expression of a subset of lncRNAs in HCC cells. Among these lncRNAs, the expression levels of HNF1A-AS1 were notably correlated with HNF1α levels in HCC cells and human HCC tissues. HNF1α activated the transcription of HNF1A-AS1 by directly binding to its promoter region. HNF1A-AS1 inhibited the growth and the metastasis of HCC cells in vitro and in vivo. Moreover, knockdown of HNF1A-AS1 reversed the suppressive effects of HNF1α on the migration and invasion of HCC cells. Importantly, HNF1A-AS1 directly bound to the C-terminal of SHP-1 with a high binding affinity (KD = 59.57 ± 14.29 nM) and increased the phosphatase activity of SHP-1. Inhibition of SHP-1 enzymatic activity substantially reversed the HNF1α- or HNF1A-AS1-induced reduction on the metastatic property of HCC cells.

**Conclusions:**

Our data revealed that HNF1A-AS1 is a direct transactivation target of HNF1α in HCC cells and involved in the anti-HCC effect of HNF1α. HNF1A-AS1 functions as phosphatase activator through the direct interaction with SHP-1. These findings suggest that regulation of the HNF1α/HNF1A-AS1/SHP-1 axis may have beneficial effects in the treatment of HCC.

**Electronic supplementary material:**

The online version of this article (10.1186/s12943-018-0813-1) contains supplementary material, which is available to authorized users.

## Background

Hepatocellular carcinoma (HCC) is one of the most common cancers and the second leading cause of cancer mortality worldwide [1]. Recently, accumulating evidences have demonstrated that long non-coding RNAs (lncRNAs), a large class of transcripts longer than 200 nucleotides (nt) without protein-coding potentials, are closely associated with the occurrence and development of human cancers, including HCC [2–5] .

Hepatocyte nuclear factor 1α (HNF1α), a POU-homeodomain family transcription factor, expressed predominantly in the liver, and it regulates many aspects of hepatocyte functions [6–8]. We have previously reported that the enforced expression of HNF1α impedes the growth of HCC xenografts in mice by inducing the differentiation of hepatoma cells into hepatocytes [9]. Our recent study further demonstrated that hepatocyte-specific Hnf1α knockout mice spontaneously develop HCC from fatty liver without cirrhosis [10]. In addition, it has been reported that HNF1α inhibits Wnt and NF-κB signalling during hepatocarcinogenesis and HCC metastasis by transcriptionally regulating the expression of miR-194 [11, 12]. However, whether lncRNAs contribute to the suppressive effect of HNF1α on HCC remains unclear.

Src homology region 2 (SH2) domain-containing phosphatase 1 (SHP-1, also known as PTPN6), a non-receptor protein tyrosine phosphatase (PTP), is predominantly expressed in haematopoietic and epithelial cell and widely accepted as a negative regulator of inflammation and as a tumour suppressor [13, 14]. SHP-1 plays a crucial role in glucose homeostasis and lipid metabolism in the liver [15–17]. Previous studies indicated that sorafenib, a multi-kinase inhibitor approved for HCC treatment, increased the activity of SHP-1 in HCC [18–20]. SHP-1 also repressed TGF-β-induced EMT and further inhibited the migration and invasion of HCC cells [21]. Our recent study revealed that HNF1α inhibits liver fibrosis by regulating SHP-1 expression in rat hepatocytes [22]. Therefore, it is of interest to clarify the role of SHP-1 in anti-tumour effect of HNF1α.

In this study, we reported that HNF1A-AS1, an lncRNA found only in primates, was transcriptionally activated by HNF1α in human HCC cells. HNF1A-AS1 inhibited the malignant properties of HCC cells both in vitro and in vivo and contributed to the anti-tumour effects of HNF1α. Importantly, we found that HNF1A-AS1 mediated the regulation of HNF1α on SHP-1 activity in HCC cells and increased the phosphatase activity of SHP-1 by directly binding to the C-terminal of SHP-1. Blocking the SHP-1 activity reverse the anti-HCC effect of HNF1α and HNF1A-AS1. These findings suggested that HNF1A-AS1 exerts its suppressing effect on HCC through direct regulating the enzyme activity of SHP-1.

## Methods

### Viruses

To generate lentiviruses for the overexpression of HNF1α and HNF1A-AS1, full-length cDNA of HNF1α and HNF1A-AS1 were cloned into the pCDH-CMV-MCS-EF1-copGFP vector (System Biosciences). For HNF1α-targeting short hairpin RNA expression, oligonucleotides encoding HNF1α short hairpin RNA (GATCCGGTCTTCACCTCAGACACTTTC AAGAGAAGTGTCTGAGGTGAAGACCTTTTTG) were cloned into the pmiRZIP vector (System Biosciences). All vectors were verified by sequencing. The primer sequences are listed in Additional file 1: Table S1.

The lentiviral vectors were transfected into subconfluent HEK293T cells together with the packaging plasmid psPAX2 and envelope plasmid pMD2.G (Addgene) using FuGENE 6 transfection reagent (Promega) to produce lentiviral particles. The lentiviruses in the medium were collected 48 h later and concentrated by ultracentrifugation.

### Cell culture

The human HCC cell lines Huh-7, MHCC-97 L, MHCC-97H, MHCC-LM3, SMMC-7721 and YY-8103 were obtained from Type Culture Collection of the Chinese Academy of Sciences (Shanghai, China). HepG2, Hep3B, PLC/PRF/5 and 293 T cells were from American Type Culture Collection. HepG2 and Hep3B cells were cultured in Eagle’s minimum essential medium (MEM) supplemented with 10% FBS and 1 × nonessential amino acid (NEAA). The other cells were cultured in Dulbecco’s modified Eagle’s medium (DMEM) supplemented with 10% FBS.

### Microarray analysis

Total RNA was extracted from Huh-7 cells infected with Lenti-HNF1α, Lenti-shHNF1α and their corresponding control viruses and subjected to hybridization on an Agilent Human 180 K lncRNA microarray v4.0 (Agilent, Santa Clara, CA, USA) according to the manufacturer’s instructions. Images of hybridized microarrays were scanned using an Agilent Scanner (Agilent), and the raw data were normalized using a quantile algorithm, Gene Spring Software 11.0 (Agilent). Microarray hybridization, scanning and analysis were performed by Shanghai Biotechnology Corporation (Shanghai, China). The differentially expressed lncRNAs were acquired according to the significance of a false discovery rate (FDR) at 5% and a fold-change (FC) cut-off at 2. The entire dataset is available at NCBI Gene Expression Omnibus (http://www.ncbi.nlm.nih.gov/geo/) under the accession number GSE103128.

### Human tissues

All human HCC samples were obtained from HCC patients undergoing surgical resection at the Eastern Hepatobiliary Surgery Hospital (Shanghai, China). Written informed consent was obtained from all patients. All human experiments were approved by the Ethics Committee of the Second Military Medical University (Shanghai, China).

### Total RNA isolation and real-time polymerase chain reaction (RT-PCR)

Total RNA was isolated from cells or tissues with ready-to-use TRIzol Reagent (TaKaRa). SuperScript III reverse transcriptase (Invitrogen) was employed to synthetize first-strand cDNA. Real-time PCR was performed in an ABI StepOne Real-time Detection System (Life Technologies) using SYBR Green (Takara). The primer sequences are listed in Additional file 1: Table S1.

### Western blotting analysis

Proteins were extracted using RIPA buffer (P0013B, Beyotime, Suzhou, China) supplemented with protease inhibitor cocktail (Roche), separated via sodium dodecylsulfate-polyacrylamide gel electrophoresis (SDS-PAGE), and transferred to NC membranes (HAHY00010, Millipore). The membranes were blocked in PBS-T containing 5% skim milk or BSA for 2 h and then incubated with primary antibodies overnight at 4 °C. After 2 h of incubation with secondary antibody (donkey anti-mouse or donkey anti-rabbit, IRDye 700 or IRDye 800), signals were detected using an Odyssey Infrared Imaging System (LI-COR) at 700 or 800 nm.

### Northern blotting and rapid amplification of cDNA ends (RACE)

Northern blotting for HNF1A-AS1 was performed on purified polyA+ RNA using biotin-labelled probes. Briefly, polyA+ RNA was purified using the Dynabeads® mRNA DIRECT Purification Kit (Thermo Fisher). Then, 10 μg polyA+ RNA was electrophoresed and transferred to a positively charged Biodyne B Nylon Membrane (Pall, P/N 60200). The transferred RNA was then fixed to the nylon membrane using UV cross-linking. RNA was detected with a specific oligonucleotide probe representing HNF1A-AS1 labelled with biotin-16-dUTP (Roche). Human Liver Marathon-Ready cDNA (Clontech) was used to perform 3′ and 5′ RACE according to the manufacturer’s instructions. All the primer sequences are listed in Additional file 1: Table S1.

### Chromatin immunoprecipitation (ChIP) assay

Huh-7 cells were cross-linked and sonicated to shear DNA to an average fragment size of 200 to 1000 bp. For endogenous chromatin immunoprecipitation, the chromatin fragments were immunoprecipitated using 10 μg anti-HNF1α antibodies (sc-10,791, Santa Cruz). Normal rabbit IgG was used as a negative control. For chromatin immunoprecipitation using ectopic HNF1α, chromatin fragments derived from Huh-7 cells transfected with Flag-HNF1α or Flag-CMV-2 were immunoprecipitated with anti-FLAG Ab-conjugated agarose beads (A2220; Sigma-Aldrich, St. Louis, MO). DNA extraction was performed using Qiagen Purification Kits. Real-time PCR analysis was carried out to detect HNF1α binding sites on the HNF1A-AS1 promoter. OCT1 was used as a positive control. The primer sequences for ChIP-PCR are shown in Additional file 1: Table S1.

### Luciferase reporter assay

To test the transcriptional activity of HNF1α on the HNF1A-AS1 promoter, an HNF1A-AS1 promoter fragment containing the HNF1α response element (RE) was amplified by PCR from genomic DNA and cloned into the pGL3-Promoter vector (E1761, Promega). The HNF1α-RE was mutated using the Hieff MutSite-Directed Mutagenesis Kit (Yeasen Biotechnology, Shanghai, China). Huh-7 cells pre-infected with Lenti-HNF1α for 24 h were co-transfected with HNF1α-RE-LUC vectors together with the control pRL-SV40 vector (E2261, Promega). Luciferase activity was measured using the Dual-Glo Luciferase Assay System (E2920, Promega) 48 h post-transfection. All constructs were verified by DNA sequencing. The primer sequences for the constructs are listed in Additional file 1: Table S1. At least three independent transfection experiments were carried out for each condition.

### Cell proliferation, colony formation, and soft agar colony formation assay

HCC cells were infected with lentiviruses and adenoviruses or transfected with siRNAs for 8–12 h and then plated in 96-well plates at the density of 3000 cells per well with 100 μl of complete culture medium. Cell proliferation was examined by the Cell Counting Kit-8 (Dojindo, Tokyo, Japan) according to the manufacturer’s instructions. For colony formation assays in culture plates, HCC cells infected or transfected for 24–48 h were seeded on 60 mm dishes. For soft agar colony formation assays, HCC cells infected or transfected for 24–48 h were resuspended in medium containing 0.5% low melting point agarose and seeded in plates containing medium with 1% solidified agarose. After 2 to 3 weeks, colonies on plates or in soft agar were stained with 0.1% crystal violet, photographed and counted. At least three independent experiments were performed for each condition.

### In vitro migration and invasion assay

In vitro migration and invasion assays were performed as described previously [23]. To investigate the effect of SHP-1 on the role of HNF1α and HNF1A-AS1 in HCC cells, 15 μM/L PTP inhibitor III (Merck Millipore) was added to the medium, and DMSO was used as control. After incubation for 24–48 h at 37 °C, the cells were fixed and stained as previously described [23]. Image analysis software (Image-Pro Plus 6.0, Media Cybernetics) was used to measure the area of positive staining.

### Animal models

Male BALB/c nude mice (5~ 6 weeks old) or NOD/SCID mice (5~ 6 weeks old) were purchased from Shanghai Experimental Animal Center of the Chinese Academy of Sciences, Shanghai, China. To detect the effect of HNF1A-AS1 on the tumourigenicity of HCC cells, 2 × 10^6^ Huh-7 or 1 × 10^6^ MHCC-LM3 cells pre-infected with Lenti-HNF1A-AS1 or control virus were subcutaneously injected into flanks of BALB/c nude mice. Tumour formation was estimated as previously described [24]. MHCC-LM3 cells stably expressing luciferase and infected with Lenti-HNF1A-AS1 or control virus were injected via the tail veins into NOD/SCID mice to generate a tumour metastasis model. Mice were monitored using the IVIS 200 imaging system (Caliper Life Sciences, Hopkinton, MA) once per week and sacrificed 6 weeks after injection. Metastatic tumour nodules in different organs of the mice were further monitored using in vivo luciferase assays [25]. All animal experiments were performed in accordance with protocols approved by the Institutional Animal Care and Use Committee at the Second Military Medical University, Shanghai, China.

### Protein recombination and purification

Recombinant His-SHP-1, GST-SHP-1 and GST-SHP-1 variants were expressed using pET28a or pGEX4T-1 expression vectors in Escherichia coli. Recombinant His-SHP-1 was purified using a nickel affinity chromatography column (5 mL HisTrap FF, GE Healthcare). GST-SHP-1 and GST-SHP-1 variants were purified with Glutathione SepharoseTM 4B–beads (GE Healthcare).

### RNA immunoprecipitation (RIP) assay

RNA-binding protein immunoprecipitation (RIP) assays were performed as previously described with minor modifications [24]. Briefly, primary antibody against SHP-1 (sc-287 Santa Cruz) was used to immuneprecipitate the endogenous SHP-1; anti-FLAG Ab-conjugated agarose beads (Sigma-Aldrich) were used for immuneprecipitating Flag-SHP-1 in Huh-7 cells transfected with pFlag-CMV-SHP-1 or Flag-CMV-SHP-1Δ517–597.

### RNA pulldown assay

Biotin-labelled RNA was transcribed in vitro with the Biotin RNA Labelling Mix (Roche) and T7 RNA polymerase (Ambion) and purified using the RNeasy Mini Kit (ZOMY). To allow proper secondary structure formation, biotinylated RNA in RNA structure buffer (10 mM Tris pH = 7.0, 0.1 M KCl, 10 mM MgCl_2_) were heated to 90 °C for 2 min, put on ice for 3 min, and left at room temperature (RT) for 30 min. The folded RNA was mixed with 1 mg Huh-7 cell lysate or 1 μg recombinant His-SHP-1 in 500 μl RIP buffer and incubated at RT for one hour. The RNA-protein complexes were captured with 25 μl washed streptavidin agarose beads (Invitrogen) at RT for one hour. The beads were briefly washed five times with RIP buffer (50 mM Tris pH = 7.4, 150 mM NaCl, 2 mM MgCl_2_, 0.5% NP40) and boiled in SDS buffer. The retrieved proteins were detected by western blotting.

### Bio-layer interferometry (BLI) assay

A ForteBio Octet® K2 System (Pall) was used to measure the binding kinetics of HNF1A-AS1 with purified His-SHP1. All of the assays were performed at 30 °C in opaque flat-bottom 96-well plates (Greiner), with agitation set to 1000 rpm in DEPC-treated PBS (pH = 7.4) supplemented with 0.01% Tween-20 to minimize nonspecific interactions. Biotinylated HNF1A-AS1 (100 nM) in PBS-Tween were immobilized on super streptavidin-coated biosensors (Pall) for 300 s. The biosensor tips were equilibrated in buffer for 600 s prior to binding purified His-SHP1 and His-SHP1Δ517–597 at increasing concentrations (0 nM, 31.25 nM, 62.5 nM, 125 nM, 250 nM, and 500 nM) for 300 s. The complex was allowed to dissociate in PBS-Tween for 600 s. Data were analysed using Octet software, version 9.0 (Pall). An empty sensor was used to bind protein at high concentration (1000 nM) as the negative control. Experimental data were fitted into binding equations describing a 1:1 interaction. Global analyses of the datasets assuming that binding was reversible (full dissociation) were carried out using nonlinear least-squares fitting, allowing a single set of binding parameters to be simultaneously obtained for all concentrations used in each experiment.

### Phosphatase assay

A RediPlate 96 EnzChek® Tyrosine Phosphatase Assay Kit (R-22067) was used for SHP-1 activity assay (Molecular Probes, Invitrogen, CA). To detect the phosphatase activity of endogenous SHP-1, SHP-1 protein in Huh-7 cell lysates was incubated with anti-SHP-1 antibody at 4 °C overnight and precipitated with Protein G-Agarose beads (Roche). The beads were washed with immunoprecipitation buffer (20 mM Tris-HCl (pH 8.0), 50 mM NaCl, 0.5% Triton-X-100, and 10% glycerol) and placed into RediPlate wells. SHP-1 activity was measured at 360/40 and 460/40 nm. The amount of SHP-1 protein used for the phosphatase assay were evaluated by western blotting. For in vitro phosphatase assay, 4 nM recombinant His-SHP-1 or His-SHP-1Δ517–597 protein and 4 nM HNF1A-AS1 were added into RediPlate wells and incubated for 30 min at RT before reading fluorescence.

### Statistical analysis

Data analyses were performed with Prism 5 (GraphPad software, La Jolla, CA). For experiments involving only two groups, data were analysed with Student’s unpaired t tests. All data are presented as the mean ± SD. Statistical significance was set at *P ≤ 0.05, **P ≤ 0.01, and ***P ≤ 0.001. *P* ≤ 0.05 was considered statistically significant.

## Results

### HNF1α regulates the expression of HNF1A-AS1

**Fig. 1:**
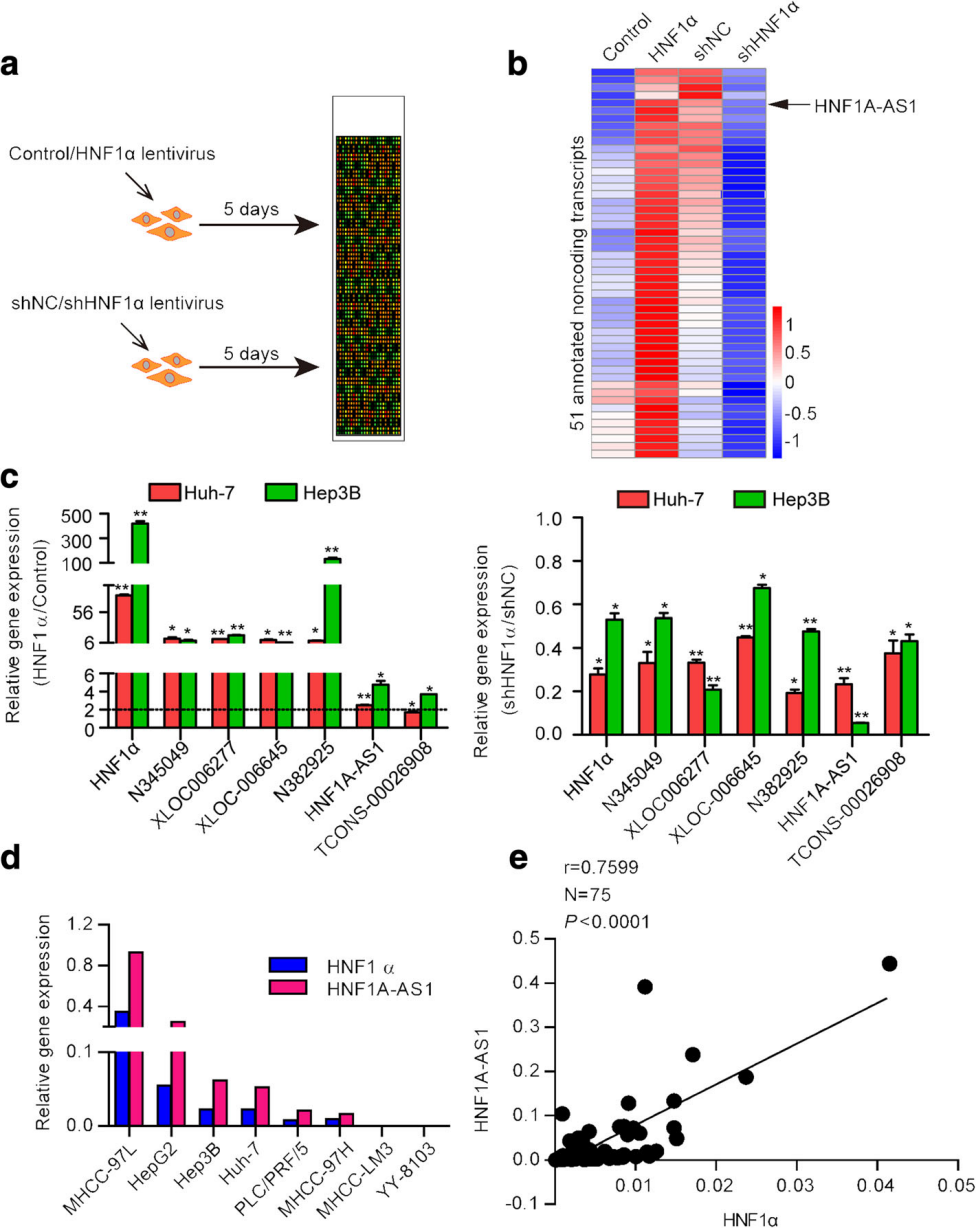
HNF1α upregulates the expression of HNF1A-AS1. **a** Experimental design for detecting HNF1α-regulated genes. Huh-7 cells infected with Lenti-HNF1α and Lenti-Control or Lenti-shHNF1α and Lenti-shNC. Total RNA was extracted from cells and subjected to microarray analysis 5 days after infection. **b** A heatmap of 51 annotated lincRNAs upregulated by HNF1α in Huh-7 cells. Colours represent the higher (red) or lower (blue) expression of transcripts, the global median scaled to 2-fold activation or repression, respectively (*P* ≤ 0.05). Black arrow denoted HNF1A-AS1. **c** Expression levels of the six selected lncRNAs were examined using real-time PCR in Huh-7 cells infected with lentivirus encoding HNF1α (left) or shHNF1α (right). **d** Expression levels of HNF1A-AS1 in different HCC cells. **e** Pearson’s correlation analysis showed positive correlation between the expression levels of HNF1A-AS1 and HNF1α in HCC tissues (r = 0.7599, *P* ≤ 0.0001, *n* = 75)

To identify the lncRNAs regulated by HNF1α, we conducted two sets of microarray analyses to obtain lncRNA expression profiles in Huh-7 cells in which HNF1α was either up- or down-regulated (Fig. 1a and Additional file 1: Figure S1a). We found that overexpression of HNF1α resulted in the upregulation of 2323 lncRNAs and the downregulation of 2713 lncRNAs in Huh-7 cells (Additional file 1: Figure S1b). In contrast, knockdown of HNF1α led to the increased expression of 2455 lncRNAs and the decreased expression of 2683 lncRNAs (Additional file 1: Figure S1b). Overlapping the two sets of data revealed that 443 lncRNAs were upregulated and 450 lncRNAs were downregulated by HNF1α in Huh-7 cells (Additional file 1: Figure S1c).

As HNF1α is a transactivation factor in hepatocytes, we selected 51 annotated lncRNAs from the 443 HNF1α-upregulated lncRNAs and analysed their promoters (Fig. 1b and Additional file 2: Table S2). Six long intergenic non-coding RNAs (lincRNAs) containing putative HNF1α-REs in their promoters were selected as the potential targets of HNF1α (Additional file 1: Table S3). Real-time PCR confirmed that HNF1α elevated the levels of these lincRNAs in HCC cells (Fig. 1c). Among these six lincRNAs, the expression level of HNF1A-AS1 was remarkably correlated with HNF1α expression in HCC cells (Fig. 1d, Additional file 1: Figure S1d and e). Moreover, a significantly positive correlation between HNF1α and HNF1A-AS1 was also detected in human HCC samples (Fig. 1e). We then analysed the characteristics of HNF1A-AS1 and further investigated the regulatory effect of HNF1α on HNF1A-AS1 in HCC cells.

### HNF1α activates the transcription of HNF1A-AS1

**Fig. 2:**
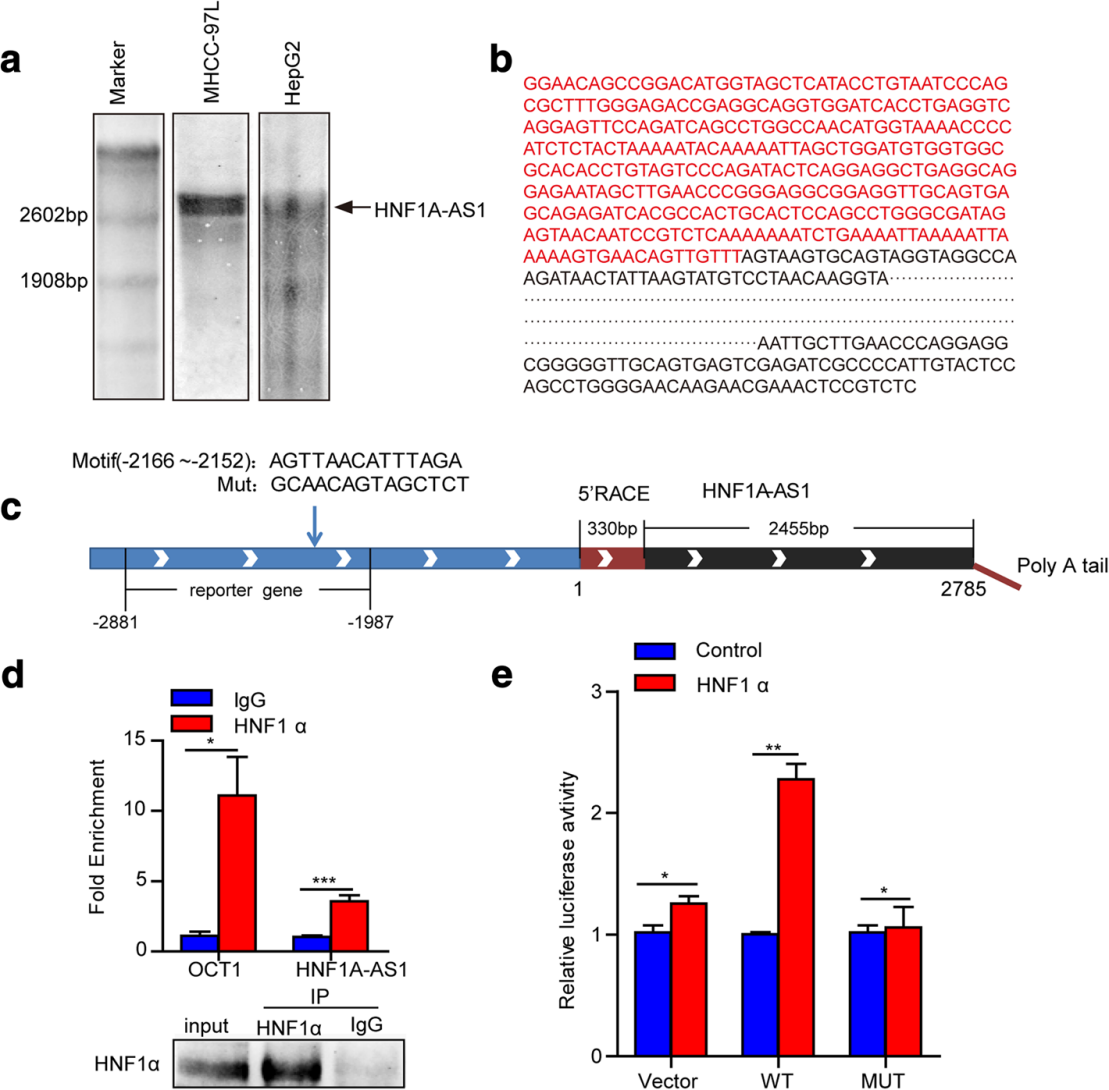
HNF1α activates the transcription of HNF1A-AS1 by directly binding to its promoter. **a** Northern blotting was performed to verify the expression of HNF1A-AS1 in MHCC-L and HepG2 cells. **b** Sequence of full-length human HNF1A-AS1 in the liver. Nucleotides identified by RACE are indicated in red. **c** Schematic representation of the full-length HNF1A-AS1 gene, the predicted HNF1α-RE in the promoter region, the mutated sequence of HNF1α-RE and the fragment for the reporter plasmid. **d** ChIP assays revealed the binding of HNF1α to the promoter region of HNF1A-AS1. Real-time PCR (upper panel) was performed to examine DNA fragments immunoprecipitated by anti-HNF1α antibody in Huh-7 cells. Western blotting (lower panel) was used to verify the immunoprecipitation of the HNF1α protein. Human OCT1 promoter containing a consensus HNF1α response element was used as the positive control. **e** Luciferase reporter assays performed with a reporter plasmid carrying the HNF1α-RE in Huh-7 cells co-transfected with Flag-CMV or Flag-HNF1α. Mutation of HNF1α-RE abolished the transcriptional activity of HNF1α. Data represent the mean ± SD. *P ≤ 0.05, **P ≤ 0.01, and ***P ≤ 0.001

The HNF1A-AS1 gene is located on human chromosome 12q24.31 and approximately 6 kb away from the HNF1α gene. It had been reported that HNF1A-AS1 is transcribed as a 2455 nt lncRNA in the opposite direction to the HNF1α gene. However, northern blotting indicated that HNF1A-AS1 is longer than 2600 nt in human hepatoma cells (Fig. 2a). We then performed 5′ rapid amplification of cDNA ends (5′ RACE) and 3′ RACE to amplify HNF1A-AS1 transcripts in a human liver cDNA library. Sequencing of PCR products revealed that the full-length transcript of HNF1A-AS1 RNA is 2785 nt with a poly A tail, which is 330 nt longer at the 5′ end than the previously published sequence of HNF1A-AS1 in the NCBI database (Fig. 2b).

According to the JASPAR database [26], a putative HNF1α-RE is located 2166 to 2152 bp upstream of the HNF1A-AS1 transcriptional start site (Fig. 2c). ChIP assays confirmed the direct binding of HNF1α to the promoter of HNF1A-AS1 (Fig. 2d, and Additional file 1: Figure S2a-c). Luciferase reporter assays also showed that the ectopic expression of HNF1α increased the transcriptional activity of the HNF1A-AS1 promoter, which was impaired by mutations in the HNF1α-RE (Fig. 2e). Taken together, these data suggested that HNF1α regulates HNF1A-AS1 by directly binding to its promoter region.

### HNF1A-AS1 suppresses the malignant properties of HCC cells

**Fig. 3:**
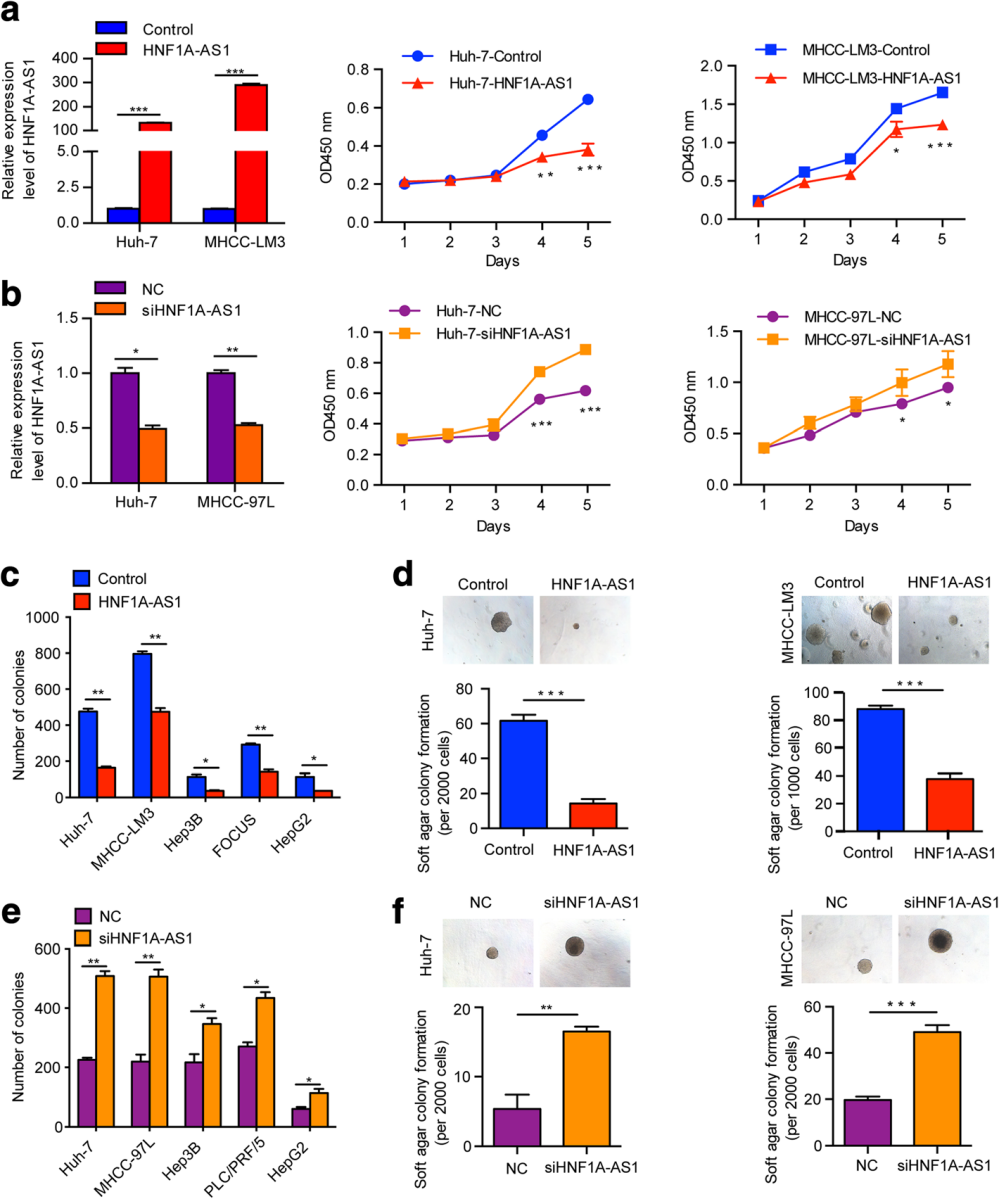
HNF1A-AS1 suppresses the proliferation and colony formation of HCC cells in vitro. **a**, **b** The expression of HNF1A-AS1 in Huh-7 cells infected with lentivirus (**a**) or transfected with siRNA (**b**) were detected by real-time PCR, respectively. Cell proliferation was measured using the Cell Counting Kit-8 (CCK8). (**c**) Colony formation assays in culture plates were performed with HCC cells infected with lentiviruses. The number of HCC cell colonies with the forced expression of HNF1A-AS1 were counted after 3 weeks. **d** Soft agar colony formation assays were performed in HCC cells treated with Lenti-HNF1A-AS1. **e**, **f** Colony formation assays (**e**) and soft agar colony formation assays (**f**) were performed in HCC cells transfected with siRNA. Data represent the mean ± SD of triplicate experiments. *P ≤ 0.05, **P ≤ 0.01, and ***P ≤ 0.001

**Fig. 4:**
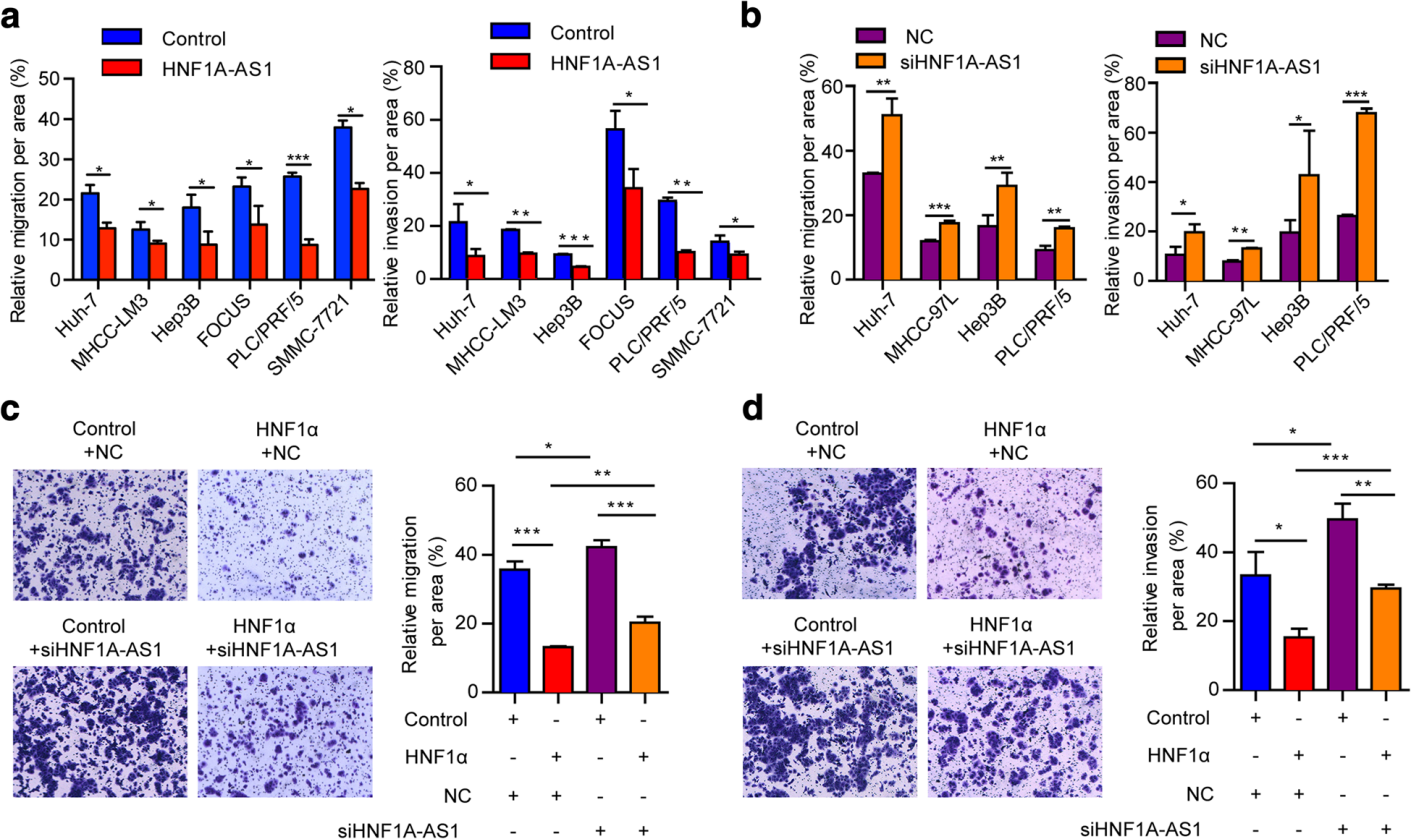
HNF1A-AS1 suppresses migration and invasion of HCC cells in vitro. **a** Migration and invasion of HCC cells infected with Lenti-HNF1A-AS1 for 72 h. **b** Cell migration and invasion of HCC cells transfected with siHNF1A-AS1 for 48 h. **c**, **d** Inhibitory effects of HNF1α on the migration (**c**) and invasion (**d**) of Huh-7 cells were reversed by knockdown of HNF1A-AS1. Data represent the mean ± SD of triplicate experiments. *P ≤ 0.05, **P ≤ 0.01, and ***P ≤ 0.001

To evaluate the potential role of HNF1A-AS1 in HCC, we modulated its expression in HCC cells. The enforced expression of HNF1A-AS1 by lentiviral infection notably suppressed HCC cell growth (Fig. 3a and Additional file 1: Figure S3a), whereas reduced HNF1A-AS1 expression via siRNA promoted the proliferation of HCC cells (Fig. 3b and Additional file 1: Figure S3b). Additionally, HNF1A-AS1 overexpression strikingly reduced both anchorage-dependent and independent colony formation of HCC cells while HNF1A-AS1 inhibition exerted the opposite effects (Fig. 3c-f, Additional file 1: Figure S3c and d). Moreover, overexpression of HNF1A-AS1 also significantly diminished the migration and invasion of HCC cells, whereas knockdown of HNF1A-AS1 exacerbated the metastatic potential of these cells (Fig. 4a and b). Furthermore, HNF1A-AS1 knockdown reduced the inhibitory effects of HNF1α on HCC malignancy (Fig. 4c and d), suggesting that HNF1A-AS1 plays a role in the anti-tumour effect of HNF1α.

### HNF1A-AS1 inhibits the tumorigenesis and metastasis of HCC in vivo

**Fig. 5:**
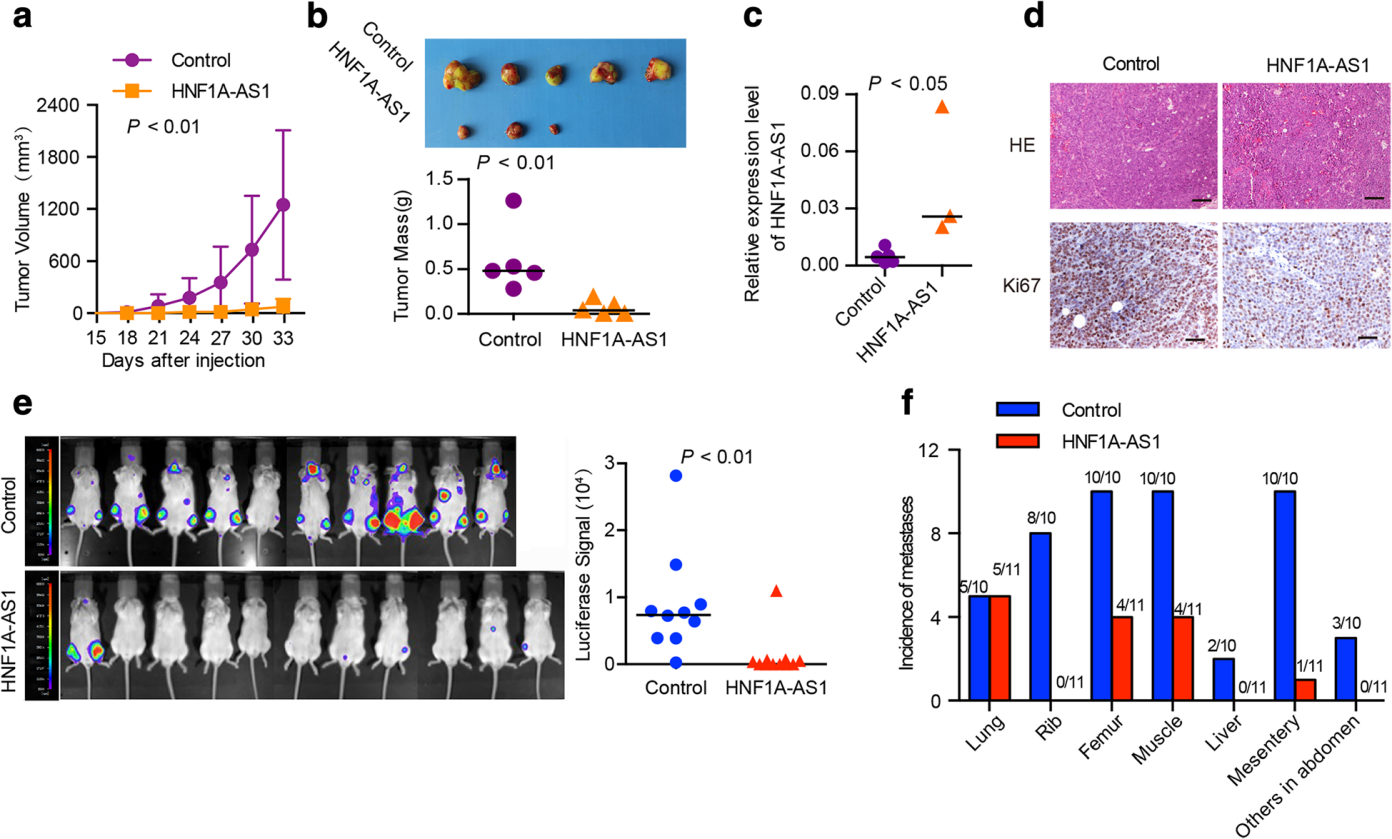
HNF1A-AS1 suppresses tumourigenicity and metastasis of HCC cells in vivo. **a** Growth curves of tumours in nude mice injected with Huh-7 cells pre-infected with Lenti-HNF1A-AS1 or control virus (*n* = 5 in each group). Data represent the mean ± SD. **b** Images (upper panel) and weight (lower panel) of xenografts derived from Huh-7 cells infected with Lenti-HNF1A-AS1 or control virus. Horizontal line indicates the median value. **c** HNF1A-AS1 expression levels in the tumour nodules. **d** HE staining showed a typical trabecular HCC pattern in both the Lenti-HNF1A-AS1 and Lenti-control groups. IHC staining showed the reduction in Ki67 expression in the Lenti-HNF1A-AS1 group compared to that in the Lenti-control group. Scale bars, 200 μm. **e** Luciferase-labelled MHCC-LM3 cells pre-infected with Lenti-HNF1A-AS1 or control virus were injected into NOD/SCID mice through the tail veins. Luciferase intensity in NOD/SCID mice indicative of metastatic nodules (left). Luciferase signals were measured by the IVIS imaging system (right). **f** Summary of metastases in different organs

To further validate the effect of HNF1A-AS1 on the tumourigenesis of HCC cells in vivo, we subcutaneously injected Huh-7 cells pre-infected with either Lenti-HNF1A-AS1 or control virus into the flanks of nude mice. Xenografts were detected on day 21 after injection in all of five mice in the control group, whereas only three mice developed xenografts in the HNF1A-AS1 group at the end of the experiment (day 33). Meanwhile, tumour nodules in the HNF1A-AS1 group were significantly smaller than those in the control group (Fig. 5a). Consistently, the tumour weight was markedly reduced in the HNF1A-AS1 group (Fig. 5b). Real-time PCR confirmed the elevated expression of HNF1A-AS1 in the Lenti-HNF1A-AS1-treated tumours (Fig. 5c). Ki67 staining revealed that HNF1A-AS1 overexpression decreased cell proliferation in the HCC xenografts (Fig. 5d). Similar results were also obtained using MHCC-LM3 cells (Additional file 1: Figure S4a-d).

We next evaluated the effect of HNF1A-AS1 on HCC metastasis in NOD/SCID mice. Luciferase-labelled MHCC-LM3 cells infected with either Lenti-HNF1A-AS1 or control virus were injected through the tail veins of mice. These mice were then subjected to in vivo luciferase assays to monitor systemic metastasis once a week for 6 weeks. As indicated by luciferase signals, the overexpression of HNF1A-AS1 markedly inhibited the metastasis of MHCC-LM3 cells in mice, especially bone and abdominal metastases (Fig. 5e). Once the mice were sacrificed, the metastatic tumour nodules in the different organs were further validated based on luciferase signals and HE staining (Additional file 1: Figure S4e). The incidence of lung metastasis was not significantly different in the two groups of mice (5/10 vs. 5/11). However, compared with that in the cells infected with control virus, the metastasis of MHCC-LM3 cells infected with Lenti-HNF1A-AS1 to the rib (8/10 vs. 0/11), femur and muscles (10/10 vs. 4/11), liver (2/10 vs. 0/11), mesentery (10/10 vs. 0/11) and other abdominal organs such as the pancreas (3/10 vs. 0/11) was markedly inhibited (Fig. 5f).

### HNF1A-AS1 directly binds to SHP-1 protein in human HCC cells

Our previous study has demonstrated the regulation of HNF1α on SHP-1 expression in rat hepatocytes [22]. In present study, we found that HNF1α activated HNF1A-AS1 expression. Then, we analyzed whether HNF1A-AS1 is involved in the regulation of SHP-1 by HNF1α in human HCC cells. However, we found that neither HNF1α nor HNF1A-AS1 was significantly correlated with the expression of SHP-1 in human HCC tissues (Additional file 1: Figure S5a and b). In addition, although HNF1α increased the expression of SHP-1 in primary rat and mouse hepatocytes (Additional file 1: Figure S6a), HNF1α or HNF1A-AS1 did not alter the levels of SHP-1 in human HCC cells (Additional file 1: Figure S6b and c), suggesting that HNF1α or HNF1A-AS1 could not regulate the expression of SHP-1.

**Fig. 6:**
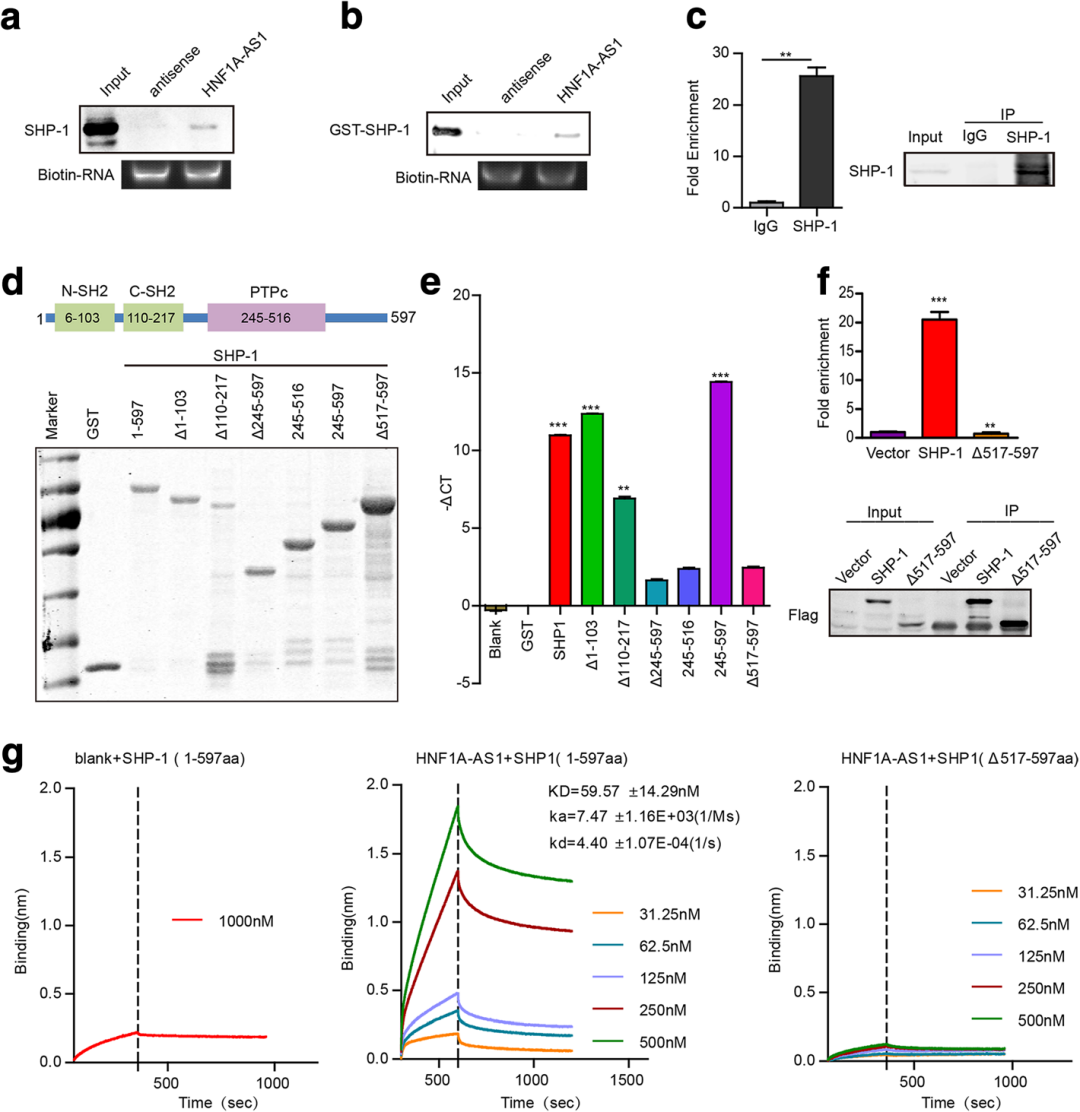
HNF1A-AS1 directly interacts with SHP-1. **a** Endogenous SHP-1 protein in Huh-7 cells was precipitated with biotin-labelled HNF1A-AS1 RNA. **b** RNA pulldown assay showed the direct interaction of HNF1A-AS1 with recombinant SHP-1 protein. **c** Association of endogenous SHP-1 and HNF1A-AS1 was detected by RIP assay. Huh-7 cell lysates were immunoprecipitated with anti-SHP-1 antibody. Immunoprecipitated HNF1A-AS1 was detected by real-time PCR. RNA from immunoprecipitation reactions with normal rabbit IgG (IgG) was used as the control. **d** Deletion mapping of the HNF1A-AS1-binding domain in SHP-1. Upper panel: Graphical illustration of the functional domains of the SHP-1 protein. Lower panel: amount of GST-SHP-1 and domain-truncated SHP-1 variants detected in the binding assay. **e** Real-time PCR was performed to detect HNF1A-AS1 binding to GST-tagged SHP-1 and domain-truncated SHP-1 variants. **f** Huh-7 cells transfected with Flag-SHP-1 or Flag-SHP-1Δ517–597. The endogenous HNF1A-AS1 co-precipitated with Flag-SHP-1 were detected by real-time PCR. Data represent the mean ± SD of triplicate experiments. **P ≤ 0.01, and ***P ≤ 0.001. **g** Bio-Layer Interferometry assay showed the binding of HNF1A-AS1 to SHP1 protein with high affinity. Deletion of 517–597 aa abolished the binding of SHP-1 with HNF1A-AS1

Interestingly, the prediction by RPISeq [27] (http://pridb.gdcb.iastate.edu/RPISeq/), a database of RNA-protein interactions, suggested a potential interaction of HNF1A-AS1 with the SHP-1 protein with high binding affinity (RF = 0.7/SVM = 0.82). We then examined the potential interaction of HNF1A-AS1 with SHP-1. RNA pull-down assays showed that HNF1A-AS1 but not its antisense transcript precipitated the SHP-1 protein in Huh-7 cells (Fig. 6a). Moreover, in vitro RNA-protein binding assay revealed that HNF1A-AS1 directly interacted with the recombinant SHP-1 protein (Fig. 6b). RIP experiments confirmed the interaction of SHP-1 with endogenous HNF1A-AS1 in Huh-7 cells (Fig. 6c). To identify the domain of SHP-1 that binds to HNF1A-AS1, truncated SHP-1 protein variants tagged with GST were expressed and purified (Fig. 6d). Protein domain mapping studies demonstrated that HNF1A-AS1 bounds to the C-terminal (517–597 aa) of the SHP-1 protein (Fig. 6e). In addition, RIP assay also demonstrated that C-terminal deletion abolished the binding of SHP-1 with HNF1A-AS1 in Huh-7 cells (Fig. 6f). Furthermore, Bio-Layer Interferometry (BLI) assay confirmed the high affinity binding of biotin-labelled HNF1A-AS1 with recombinant SHP-1 protein (KD = 59.57 ± 14.29 nM), while C-terminal deletion diminished this binding (Fig. 6g).

### HNF1A-AS1 inhibits HCC through increasing enzymatic activity of SHP-1

**Fig. 7:**
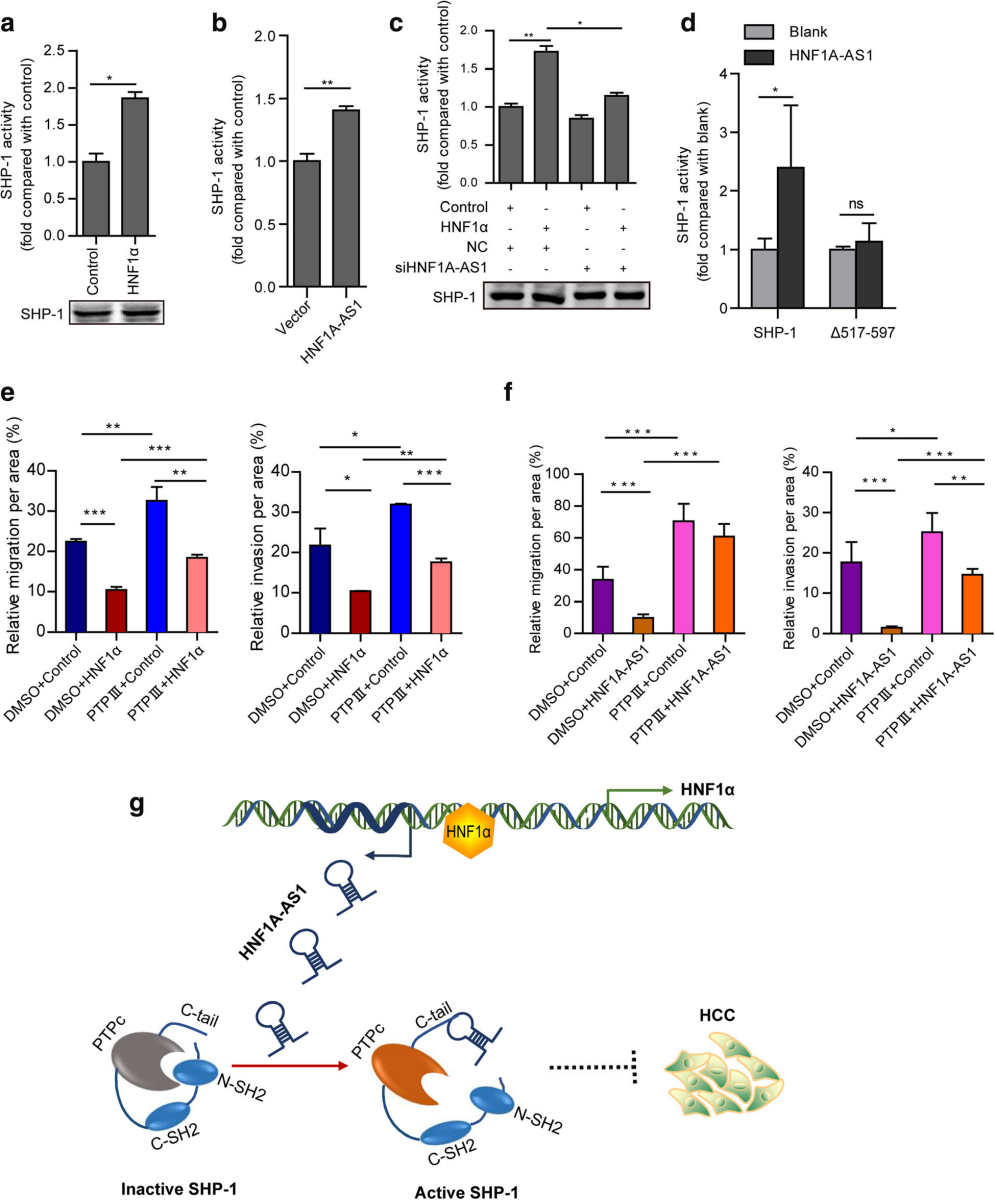
HNF1A-AS1 increases the phosphatase activity of SHP-1 by interacting with the C-terminal of SHP-1. **a** Overexpression of HNF1α increased the phosphatase activity of SHP-1 in Huh-7 cells. The amount of SHP-1 protein used for the phosphatase assay were detected by western blotting (lower panel). **b** HNF1A-AS1 enhanced the phosphatase activity of SHP-1 in Huh-7 cells. **c** Forced expression of HNF1α induced phosphatase activity of SHP-1 activation, while knockdown of HNF1A-AS1 blocked the effect of HNF1α on SHP-1. **d** HNF1A-AS1 increased the phosphatase activity of the recombinant SHP-1 protein in vitro. C-terminal deletion of SHP-1 abolished the effect of HNF1A-AS1. **e**, **f** The specific phosphatase inhibitor of SHP-1, PTP inhibitor III (PTP III), reverse the inhibitory effect of HNF1α (**e**) or HNF1A-AS1 (**f**) on the migration and invasion of Huh-7 cells. Data represent the mean ± SD of triplicate experiments. *P ≤ 0.05, **P ≤ 0.01, and ***P ≤ 0.001. **g** A schematic model representing that HNF1α increases the activity of SHP-1 in human HCC cells via upregulating HNF1A-AS1

Previous studies have demonstrated that the C-terminal region of SHP-1 is involved in the regulation of its phosphatase activity [28, 29]. We then evaluated the effect of HNF1α and HNF1A-AS1 on SHP-1 activity. Overexpression of either HNF1α or HNF1A-AS1 significantly increased the enzymatic activity of SHP-1 in Huh-7 cells (Fig. 7a and b). In addition, HNF1A-AS1 knockdown blocked HNF1α-induced SHP-1 activation (Fig. 7c), indicating that HNF1α regulates SHP-1 activity through HNF1A-AS1. Moreover, in vitro phosphatase assay showed that HNF1A-AS1 also enhanced the tyrosine phosphatase activity of recombinant SHP-1, which was abolished by the deletion of the SHP-1 C-terminal (Fig. 7d). These results suggested that HNF1A-AS1 increased SHP-1 activity through direct interaction with SHP-1. Furthermore, inhibition of SHP-1 activity using protein tyrosine phosphatase inhibitor III (PTP inhibitor III) substantially reversed HNF1α- or HNF1A-AS1-induced reduction in the migration and invasion of Huh-7 cells (Fig. 7e and f). Taken together, these data suggested that the activation of SHP-1 were involved in the anti-tumour effects of HNF1α and HNF1A-AS1.

## Discussion

It has been reported that HNF1A-AS1 is a poorly conserved lncRNA that is highly expressed in the liver, gastrointestinal track and kidney in human [30]. According to the NCBI database, HNF1A-AS1 is transcribed from a 2455-nucleotide single-exon gene. In this study, we validated the sequence of HNF1A-AS1 with RACE and northern blotting and revealed that full-length HNF1A-AS1 is 2785 nt long with poly A tail structure in human liver tissues. We also demonstrated that being an adjacent lncRNA to the HNF1α gene, HNF1A-AS1 is transcriptionally regulated by HNF1α, which is consistent with previous findings that lncRNAs are frequently regulated by their neighbouring protein-coding genes [31].

As an emerging lncRNA, the function of HNF1A-AS1 in tumours was far from being well understood. HNF1A-AS1 was upregulated in oesophageal, lung, bladder and colon cancers, and osteosarcoma but downregulated in gastric and pancreatic cancers [30, 32–37]. These contradictory results imply a tissue-specific role of HNF1A-AS1. Recently, two reports indicated that HNF1A-AS1 promoted the proliferation of HCC cells by sponging hsa-miR- 30b-5p to promote autophagy or by repressing the NKD1 and p21 via binding to EZH2 [38, 39]. However, the above studies did not characterize the full-length of HNF1A-AS1. Furthermore, the effect of HNF1A-AS1 on the metastatic property of HCC in vitro and malignancy in vivo was not reported in these papers. Therefore, the regulatory function of full-length HNF1A-AS1 in HCC still needs to be further investigated.

HNF1α has been found to play a tumour suppressor role in HCC. In this study, we found that patients with high HNF1α protein levels displayed superior overall survival (OS) by using an HCC tissue microarray containing 277 patients (median OS 42 and 33 months, respectively, *P* = 0.012; Additional file 1, Figure S7). The expression of HNF1A-AS1 was positively correlated with the expression of HNF1α in HCC tissues, implying HNF1A-AS1 may also have anti-tumour effect in HCC. In addition, our data also clearly demonstrated that the upregulation of full-length HNF1A-AS1 suppressed the proliferative and metastatic behaviours of HCC cells both in vitro and in vivo. Moreover, the knockdown of HNF1A-AS1 significantly promoted HCC malignant properties and reversed the inhibitory effects of HNF1α on HCC. These findings suggest that HNF1A-AS1 indeed acts as a tumour suppressor rather than an oncogene in HCC progression and partially mediates the anti-HCC effects of HNF1α.

It has been demonstrated that lncRNAs exert their functions by interacting with chromatin DNA, mRNAs or proteins to regulate chromatin accessibility, mRNA stability and protein activity or stability, respectively [40, 41]. Interestingly, several studies have reported that lncRNAs are also involved in the regulation of protein phosphorylation. The lncRNA BCAR4 has been reported to recruit PNUTS, a negative regulatory subunit of PP1, to H3K18ac and relieve the inhibition of RNA Pol II via the activation of the PP1 phosphatase [42]. The lncRNA NKILA has been shown to inhibit IκB phosphorylation by interacting with the NF-κB:IκB complex [43]. A recent study revealed that lncRNA TSLNC8 competitively interacted with transketolase (TKT) and STAT3 and modulated the phosphorylation of STAT3-Tyr705 and STAT3-Ser727 in HCC cells [44]. However, whether lncRNAs can directly regulate phosphatase activity has not been reported before. Here, we demonstrated that HNF1A-AS1 enhanced the activity of SHP-1 by directly binding to the C-terminal of the SHP-1 protein. Inhibition of the phosphatase activity of SHP-1 reversed the suppression of cellular migration and invasion induced by HNF1α and HNF1A-AS1. These data suggest that the increased enzymatic activity of SHP-1 contributes to the anti-tumour effects of HNF1α and HNF1A-AS1.

It is known that the expression of lncRNAs is strikingly cell type and tissue specific and in many cases, even primate specific [45, 46]. BLAST analysis using the NCBI database revealed that the HNF1A-AS1 transcript is primate-specific. No transcripts of HNF1A-AS1 have been detected in rodents to date. As a consequence of this species-specific expression pattern, we documented that HNF1α directly regulated SHP-1 expression in rodents such as mice and rats, while increased the activity of SHP-1 in human HCC cells via upregulating HNF1A-AS1. Thus, we proposed that HNF1A-AS1 orchestrated the regulatory effect of HNF1α on SHP-1 in a more delicate and complex manner in human cells (Fig. 7g).

## Conclusion

In conclusion, this study revealed that the full length of HNF1A-AS1 is 2785 nt, which is 330 bp longer than the previous reported sequence. HNF1A-AS1 is directly transcriptional regulated by HNF1α and mediates the anti-HCC effect of HNF1α in HCC cells. Moreover, we reported that HNF1A-AS1 exerts its suppressor role of HCC via interacting with SHP-1 as an enzyme activator, which extends our knowledge regarding the function of lncRNAs. These findings may imply that manipulation of HNF1A-AS1 expression might have therapeutic effects against HCC.

## Additional files

Additional file 1: **Figure S1.** Identification of HNF1α-regulated lncRNAs. **Figure S2.** HNF1α directly binds to the promoter region of HNF1A-AS1. **Figure S3.** HNF1A-AS1 suppresses the malignancy of HCC cells. **Figure S4.** Enforced expression of HNF1A-AS1 suppresses tumourigenicity and metastasis of MHCC-LM3 cells. **Figure S5.** The correlation analysis between the expression levels of SHP-1 and HNF1α or HNF1A-AS1 levels in human HCC tissues. **Figure S6.** HNF1α and HNF1A-AS1 do not regulate the expression of SHP-1 in human HCC cells. **Figure S7.** Reduction of HNF1α predicts poor prognosis of patients. **Table S1.** Oligonucleotides used in real-time PCR, cloning and knockdown studies. Primer sequences for real-time PCR. **Table S3.** Binding motif of lncRNA promoter regions for HNF1α RE. (PDF 2800 kb) (PDF 15484 kb)

Additional file 2: **Table S2.** Fifty-one annotated lncRNAs upregulated by HNF1α. (XLSX 16 kb)

## Abbreviations

ChIP: Chromatin immunoprecipitation
HCC: Hepatocellular carcinoma
HE: hematoxylin and eosin
HNF1A-AS1: HNF1A antisense RNA 1
HNF1α: Hepatocyte nuclear factor 1α
lncRNAs: Long non-coding RNAs
RIP: RNA-binding protein immunoprecipitation
SHP-1: SH2 domain-containing tyrosine phosphatase-1
