# Supplementary material for: The HNF1α-regulated lncRNA HNF1A-AS1 reverses the malignancy of hepatocellular carcinoma by enhancing the phosphatase activity of SHP-1
Source: Mol Cancer. 2018 Feb 21;17:63. doi: 10.1186/s12943-018-0813-1 (PMC5822613; doi:10.1186/s12943-018-0813-1)
Supplement: Supplementary file 1 — Figure S1. Identification of HNF1α-regulated lncRNAs. Figure S2. HNF1α directly binds to the promoter region of HNF1A-AS1. Figure S3. HNF1A-AS1 suppresses the malignancy of HCC cells. Figure S4. Enforced expression of HNF1A-AS1 suppresses tumourigenicity and metastasis of MHCC-LM3 cells. Figure S5. The correlation analysis between the expression levels of SHP-1 and HNF1α or HNF1A-AS1 levels in human HCC tissues. Figure S6. HNF1α and HNF1A-AS1 do not regulate the expression of SHP-1 in human HCC cells. Figure S7. Reduction of HNF1α predicts poor prognosis of patients. Table S1. Oligonucleotides used in real-time PCR, cloning and knockdown studies. Primer sequences for real-time PCR. Table S3. Binding motif of lncRNA promoter regions for HNF1α RE. (PDF 2800 kb) (PDF 15484 kb) [file 12943_2018_813_MOESM1_ESM.pdf]

Supplementary Information

The HNF1α-regulated lncRNA HNF1A-AS1 reverses the malignancy of hepatocellular carcinoma by enhancing the phosphatase activity of SHP-1

Chen-Hong Ding, Chuan Yin, Shi-Jie Chen, Liang-Zhi Wen, Kai Ding, Shu-Juan Lei, Jin-Pei Liu, Jian Wang, Kai-xian Chen, Hua-liang Jiang, Xin Zhang, Cheng Luo, Wei-Fen Xie

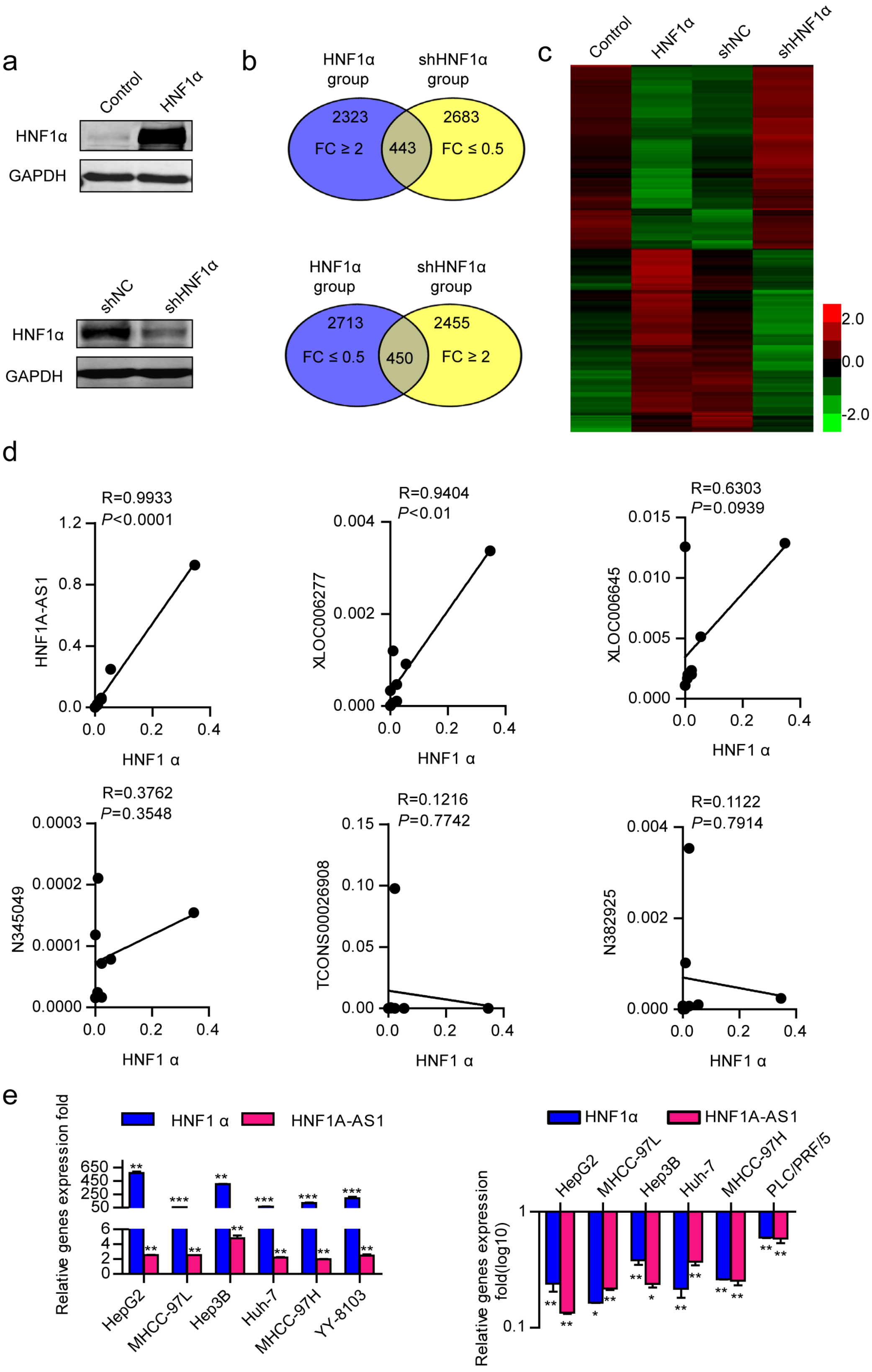

Figure S1. Identification of HNF1α-regulated lncRNAs.

(a) Expression levels of HNF1α in Huh-7 cells infected with Lenti-HNF1α and Lenti-Control

or Lenti-shHNF1α and Lenti-shNC. (b) Venn diagram depicting the differentially expressed lncRNAs in Huh-7 cells with HNF1α overexpression (HNF1α group) and HNF1α knockdown (shHNF1α group). The blue circles represent the number of lncRNAs upregulated (fold-change ≥ 2) or downregulated (fold-change ≤ 0.5) in HNF1α group. The yellow circles show the number of lncRNAs differentially expressed in shHNF1α group. FC: fold-change. (c) Heatmap representing the 443 HNF1α-upregulating lncRNAs and the 450 HNF1α-downregulating lncRNA in Huh-7 cells. Expression levels are represented in shades of red and green, indicating the expression levels above and below the median value across all the samples, respectively (2-fold, FDR<0.5). (d) Pearson's correlation analysis of the expression levels of HNF1α and the 6 selected lncRNAs in different HCC cell lines (n=8). (e) HNF1α overexpression (left) increased whereas HNF1α knockdown (right) decreased the expression of HNF1A-AS1 in HCC cells. Data represent the mean±SD.

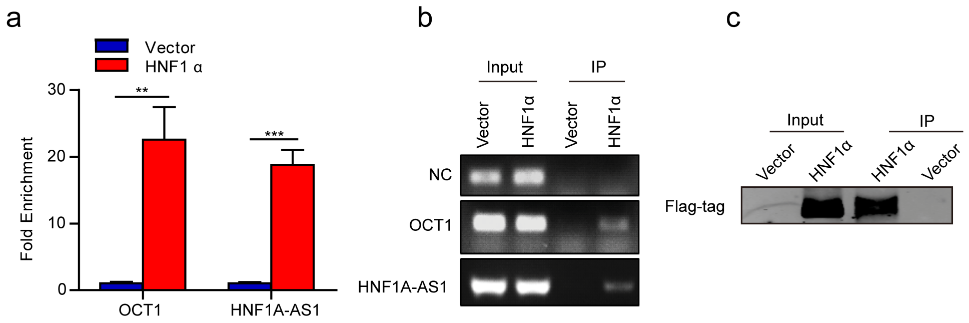

Figure S2. HNF1α directly binds to the promoter region of HNF1A-AS1.

ChIP assay performed in Huh-7 cells transfected with pCMV-Flag-HNF1α. (a,b) Real-time PCR (a) and semiquantitative PCR (b) were performed to examine the DNA fragments immunoprecipitated by anti-Flag M2 beads with primers against HNF1α-RE. (c) Western blotting analysis using anti-Flag antibody.

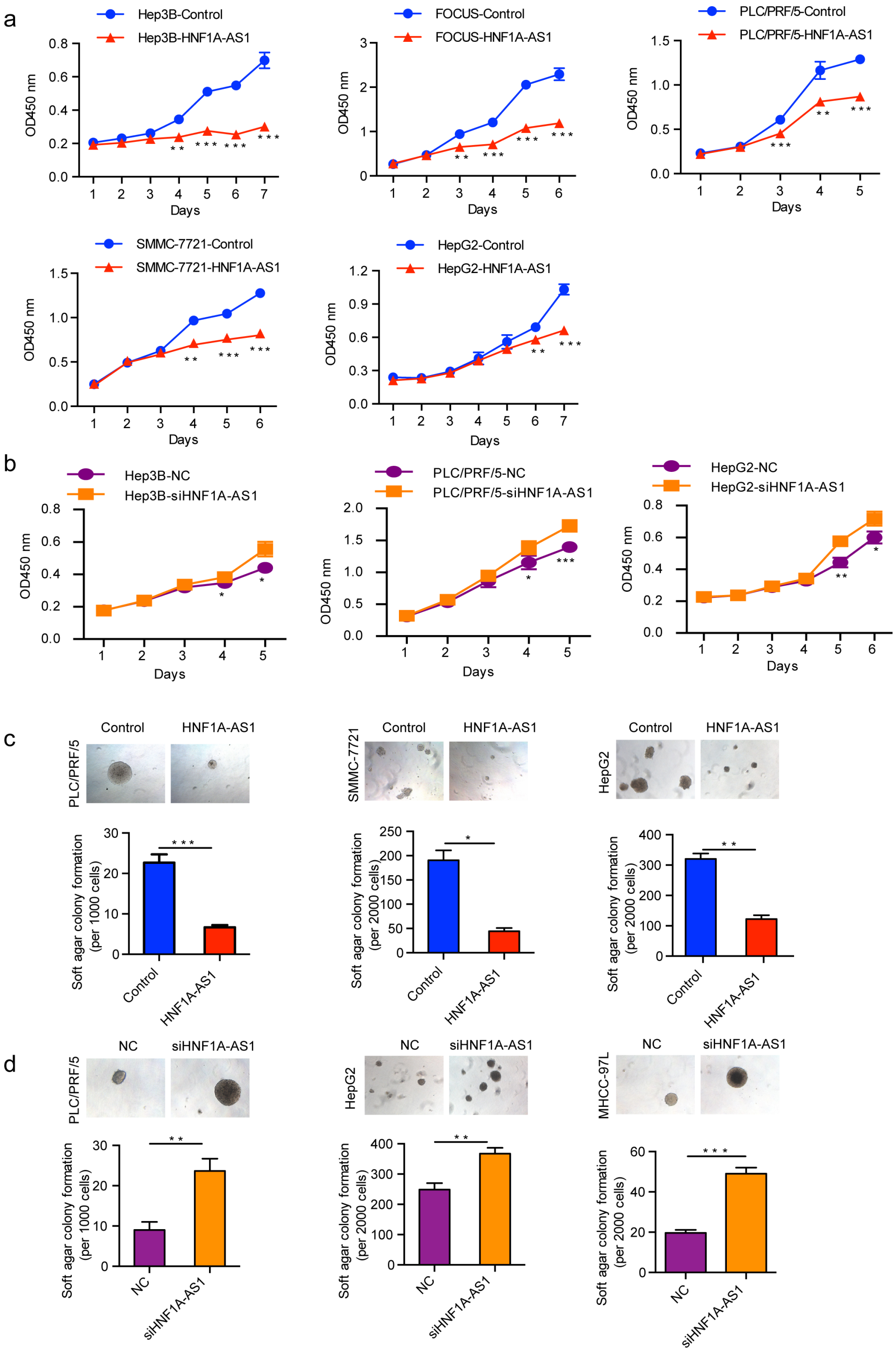

Figure S3. HNF1A-AS1 suppresses the malignancy of HCC cells.

(a,b) Proliferation was measured using the CCK-8 assay in HCC cells infected with Lenti-HNF1A-AS1 (a) or transfected with siHNF1A-AS1 (b). (c,d) Soft agar colony formation assays were performed in HCC cells treated with Lenti-HNF1A-AS1 (c) or

siHNF1A-AS1 (d).

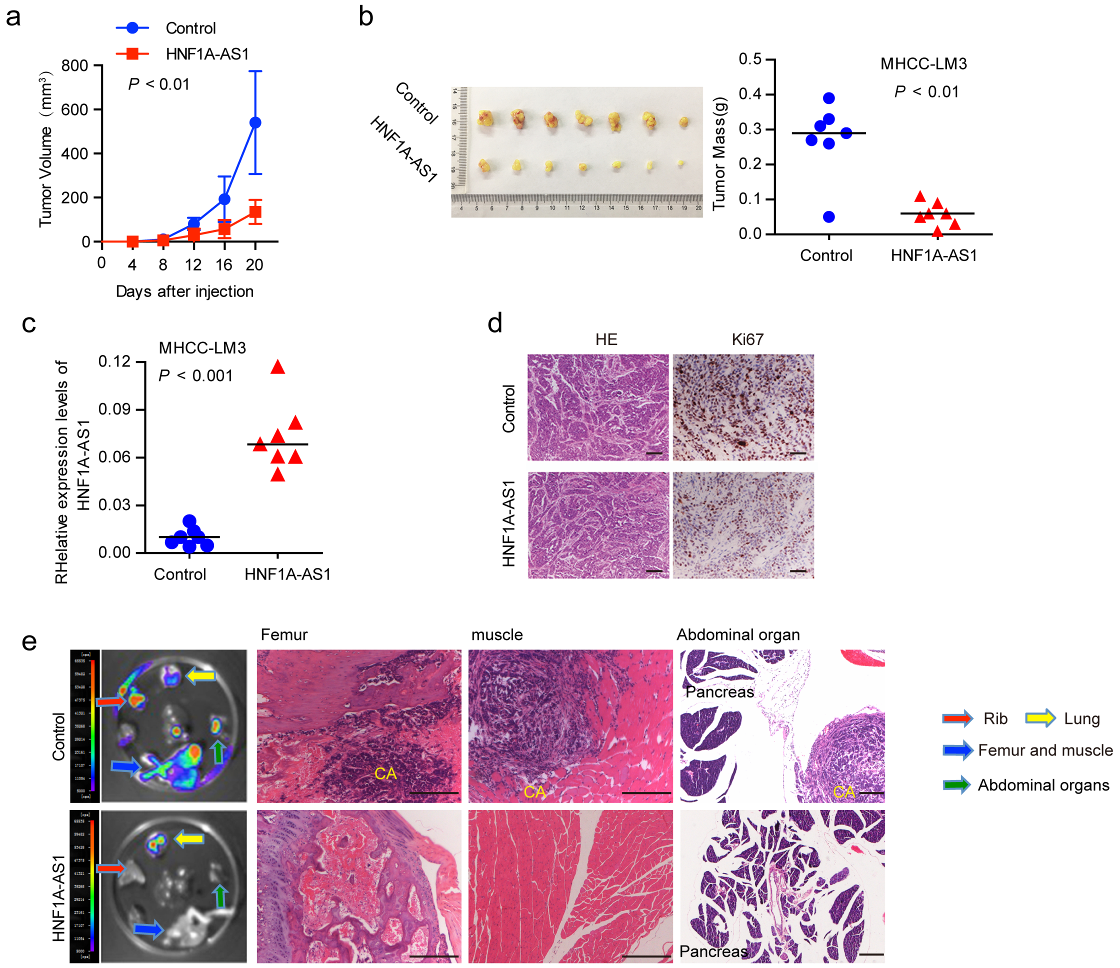

Figure S4. Enforced expression of HNF1A-AS1 suppresses tumourigenicity and metastasis of MHCC-LM3 cells.

(a) Growth curves of xenografts (n=7 in each group). (b) Images and tumour weight of the xenografts. (c) Expression levels of HNF1A-AS1 in tumour nodules. (d) HE staining and Ki67 staining of the xenografts tissues. Scale bars, 200 μm. (e) Luciferase-labelled MHCC-LM3 cells pre-infected with Lenti-HNF1A-AS1 or control virus were injected into NOD/SCID mice through the tail veins. Representative images of luciferase signals and HE staining showed metastatic nodules in different organs.

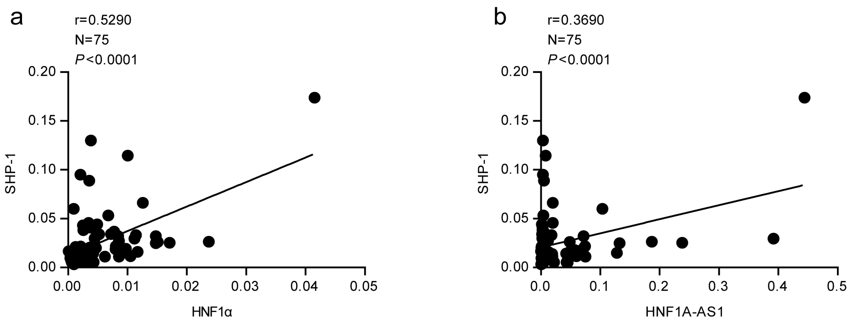

Figure S5. The correlation analysis between the expression levels of HNF1α (a) or HNF1A-AS1 (b) and SHP-1 levels in human HCC tissues.

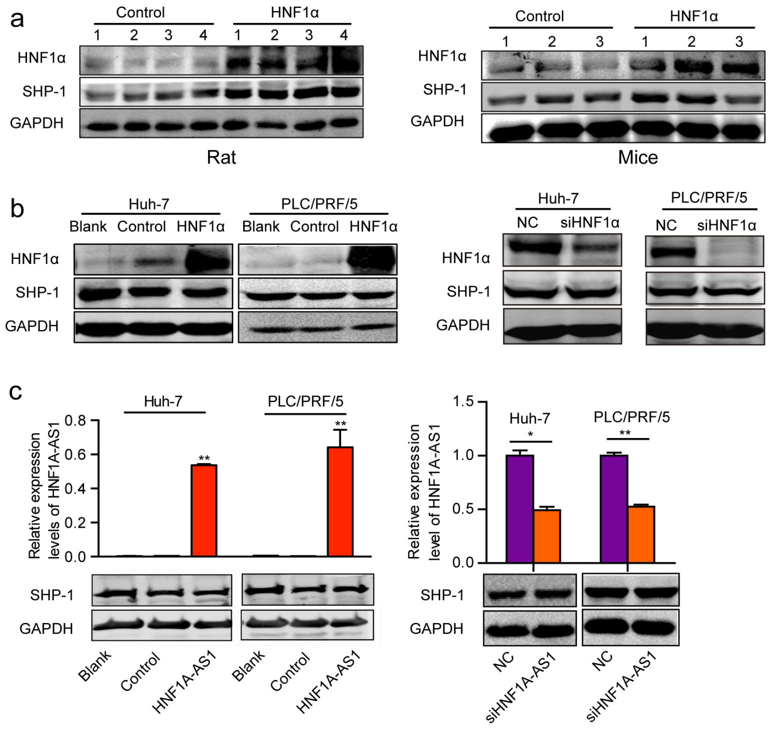

Figure S6. HNF1α and HNF1A-AS1 do not regulate the expression of SHP-1 in human HCC cells.

(a) HNF1α upregulates the expression of SHP-1 in primary rat (left) and mouse (right) hepatocytes. (b) Overexpression and knockdown of HNF1α does not affect the expression of SHP-1 in Huh-7 and PLC/PRF/5 cells. (c) HNF1A-AS1 does not regulate the expression of SHP-1 in HCC cells. Data represent the mean ± SD

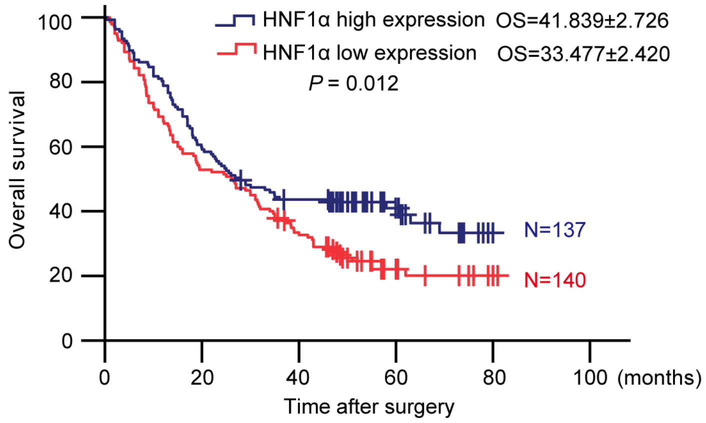

Figure S7. Reduction of HNF1α predicts poor prognosis of patients.

Tissue microarrays containing 277 HCC samples were performed to detect HNF1α protein levels. Kaplan–Meier analysis of the overall survival (OS) of 277 HCC patients. The median HNF1α protein level for all 277 HCC samples was chosen as the cut-off point. P = 0.012 by the log-rank.

Table S1. Oligonucleotides used in real-time PCR, cloning and knockdown studies. Primer sequences for real-time PCR.

Genes	Forward primer (5′- 3′)	Reverse primer (5′- 3′)
**Primers for real-time PCR**		
β-actin	CATCCTGCGTCTGGACCT	GTACTTGCGCTCAGGAGGAG
HNF1α	CCATCCTCAAAGAGCTGGAG	TGTTGTGCTGCTGCAGGTA
n382925	CTTTGGTTCAGGCACTATTA	AGAGTGGCTTTGTGAAGGT
n345049	ATAAAGGTAATGTGGGCAAGG	TCTGGAGGAGACAGGTGGC
HNF1A-AS1	CAAGAAATGGTGGCTATGA	TGGACTGAAGGACAAGGGT
XLOC006645	TATGAGATATGGAAGGACGAA	CCAATGAGATCACAGGGAG
XLOC006277	ACTTGGATTGATGGTGGTC	ACTGGCAGAACTGGTGATT
TCONS_00026908	CCTTCACTTTGGGCAATCTT	AGTTTATATTGCGGCCAAGC
**Primers for Northern blotting**		
actin	AGAAGAGCTACGAGCTGCCTGACG	TAGAAGCATTTGCGGTGGACGAT
HNF1A-AS1	TTCTCGTATGGATGTACTAACGGGA	CTCTGAGACTGGCTGAAGGGAC
**Primers for RACE**		
5’ RACE	CCTCCCGTTAGTACATCCATACGAGAAG	
3’ RACE	AAGAAAAGTTTTGGCCGGGAGCGGTG	
**Primers for ChIP assay**		
CHIP-NC	CTGGCTCGTGGGTAAGAATTGTCTC	ATGTCCAAGGCAATGCTAGGTTAAA
OCT1	GGCAGCGAGATCGAAGGACAACTGT	TCTCCTGCCTTCGGGTTTTCTCCAA
HNF1A-AS1	GAACCACTGAGAAAAAAAGC	ATAGGTGCCCACTGACAAGC

Primers for plasmid construction

Genes	5′-3′
HNF1α-Forward	CCGGAATTCCGAGCCATGGTTTCTAAACTGAG
HNF1α-Reverse	CGCGGATCCTTACTGGGAGGAAGAGGCCAT
HNF1A-AS1-Forward（2785 bp)	CCCAAGCTTGGAACAGCCGGACATGGTAG

HNF1A-AS1-Reverse (2785 bp)	CGGGGTACCGACGGAGTTTCGTTCTTGTTCC
HNF1A-AS1-enhancer-Forward (Reporter)	CCCAAGCTT CCTCACCCACCAGCCACAT
HNF1A-AS1-enhancer-Reverse (Reporter)	CCGCTCGAGAAAAGTAAAATGGAACAGGTGAAGT
**Primers for SHP-1 domain deletion plasmid construction**	
1-Forward	CCGGAATTCTGGTTTCACCGAGACCTCAGT
110-Forward	CCGGAATTCAGTGAGAGGTGGTACCATGGC
245-Forward	CCGGAATTCGGCTTCTGGGAGGAGTTTGAG
217- Reverse	CCGCTCGAGATAGTACGGCTGCCGCAGG
516- Reverse	CCGCTCGAGTTCAATGAACTGGGCGATGGC
597- Reverse	CCGCTCGAGTCACTTCCTCTTGAGGGAACCC
Δ110-217-Forward	AAGTACCCGCTGAACGGCTTCTGGGAGGAGTTTG
Δ110-217- Reverse	CTCCCAGAAGCCGTTCAGCGGGTACTTGAGG

**Sequences of siRNAs**	
NC (Human)	5′-UUCUCCGAACGUGUCACGUtt-3′
siHNF1A-AS1	5′-CACCUGCAUUCAAACUCGGACUGUUtt-3′

Table S3. Binding motif of lncRNA promoter regions for HNF1α RE

Name	Chromosome location	Source database	Binding motif	Score
HNF1A-AS1	chr12:121407641-121410095(-)	NCBI	AGTTAACATTTAGA	10.083
TCONS-00026908	chr19:15939785-15940818（+）	Broad Institute/ UCSC	ATTTAATTATTATT	10.033
XLOC-006277/n369860	chr7:142199954-142223674(+)	NONCODE/ Broad Institute	GATTAAAAATTAAT	10.646
XLOC006645/TCONS_00014006	chr7:149929650-149934873(-)	Broad Institute	GGTTAGTATATATA	9.515
n345049	chr9:70529060-70530580(+)	NONCODE	ATTTATTTATTTAC	9.439
n382925	chr16:20499023-20500981(+)	NONCODE	GGTTCACAATTACC	10.606
